# The Variety in the Common Theme of Translation Inhibition by Type II Toxin–Antitoxin Systems

**DOI:** 10.3389/fgene.2020.00262

**Published:** 2020-04-17

**Authors:** Dukas Jurėnas, Laurence Van Melderen

**Affiliations:** ^1^Laboratoire d’Ingénierie des Systèmes Macromoléculaires, Institut de Microbiologie de la Méditerranée, CNRS, Aix-Marseille Université, Marseille, France; ^2^Cellular and Molecular Microbiology, Faculté des Sciences, Université libre de Bruxelles, Gosselies, Belgium

**Keywords:** toxins, translation, persistence, programmed cell death, mobile genetic elements

## Abstract

Type II Toxin–antitoxin (TA) modules are bacterial operons that encode a toxic protein and its antidote, which form a self-regulating genetic system. Antitoxins put a halter on toxins in many ways that distinguish different types of TA modules. In type II TA modules, toxin and antitoxin are proteins that form a complex which physically sequesters the toxin, thereby preventing its toxic activity. Type II toxins inhibit various cellular processes, however, the translation process appears to be their favorite target and nearly every step of this complex process is inhibited by type II toxins. The structural features, enzymatic activities and target specificities of the different toxin families are discussed. Finally, this review emphasizes that the structural folds presented by these toxins are not restricted to type II TA toxins or to one particular cellular target, and discusses why so many of them evolved to target translation as well as the recent developments regarding the role(s) of these systems in bacterial physiology and evolution.

## Introduction

Bacterial toxin-antitoxin (TA) systems are generally composed of a toxic protein and its inhibitor. These small modules were originally discovered on plasmids in the 1980s where they were found to promote plasmid maintenance in growing bacterial populations (Karoui et al., 1983; Ogura and Hiraga, 1983; Jaffe et al., 1985; Gerdes et al., 1986; Hiraga et al., 1986). TA modules are classified into different types depending on the nature and mode of action of the antitoxins, as the toxins are always proteins. Antitoxins are either small RNAs that block translation of the toxin mRNA (type I) (Gerdes and Wagner, 2007) or sequesters the toxic protein (type III) (Fineran et al., 2009; Blower et al., 2011) or proteins that inhibit the activity of the toxin through direct interaction (type II) (Tam and Kline, 1989; Kamada et al., 2003; Takagi et al., 2005) or antagonize the toxic activity on the target, without any direct interaction with the toxins (type IV) (Brown and Shaw, 2003). This review will focus on type II systems. These elements are not only found in plasmids but also in other types of mobile genetic elements (such as phages and ICEs) as well as in chromosomes (see e.g., Anantharaman and Aravind, 2003; Pandey and Gerdes, 2005; Guglielmini and Van Melderen, 2011; Leplae et al., 2011; Ramisetty et al., 2016; Coray et al., 2017). While the roles of TAs, when located in mobile genetic elements, are reminiscent to that on plasmids, i.e., maintenance (Szekeres et al., 2007; Wozniak and Waldor, 2009; Huguet et al., 2016), the roles of chromosomally-encoded systems remains a largely debated topic in the field. These systems have been involved in the adaptation to adverse conditions and are considered to be stress response modules (Hayes and Van Melderen, 2011; Page and Peti, 2016; Harms et al., 2018), with a mainstream model proposing that TA systems are essential effectors of persistence to antibiotics (Gerdes and Maisonneuve, 2012). However, seminal papers supporting this hypothesis are now being retracted (Maisonneuve et al., 2018a, b; Germain et al., 2019) and contradictory data are being published (Harms et al., 2017; Shan et al., 2017; Goormaghtigh et al., 2018a; Pontes and Groisman, 2019). The involvement of TA systems in the persistence phenomenon was based on the observation that successive deletions of 10 TA systems in *Escherichia coli* lead to a gradual decrease of persistence frequency in the presence of lethal doses of ampicillin or ciprofloxacin (Maisonneuve et al., 2011). In subsequent studies, time-lapse microscopy experiments with *E. coli* strains containing fluorescent reporters revealed that cells that are able to recover from an ampicillin treatment (namely persister cells) are those in which the ppGpp level is high and TA systems are activated (Maisonneuve et al., 2013). Furthermore, the same group proposed that the HipBA system is the major regulator of persistence as the HipA toxin, by phosphorylating glutamyl-tRNA synthetase (see below), will trigger ppGpp production and the activation of the 10 other TA systems (Germain et al., 2015). We, along with other researchers, have identified major problems both with the *E. coli* strains and fluorescent reporters that were used in these studies (Harms et al., 2017; Goormaghtigh et al., 2018a). First, the strain deleted for the 10 TA systems is lysogenized with several copies of the Phi80 phages (Harms et al., 2017; Goormaghtigh et al., 2018a). This explains why that strain presents a lower persistence frequency to ciprofloxacin. Indeed, activation of the SOS response by fluoroquinolones will lead to lambdoid prophage activation and a strong decrease in viability. Second, the fluorescent reporters used to monitor ppGpp levels and TA activation are likely to not be functional, either forming protein aggregates or not being more fluorescent than the fluorescence background of control strains without the reporter (Goormaghtigh et al., 2018a). In an effort to solve the issue of TAs and persistence, we, along with other researchers, constructed a strain in which the 10 TA systems are deleted, devoid of any phage contaminants, and showed that this strain presents the same level of persistence to ampicillin or ofloxacin as the wild-type strain (Harms et al., 2017; Goormaghtigh et al., 2018a). Moreover, newly designed reporters monitoring the activation of the *yefM-yoeB* TA system allowed us to show that there is no correlation between persister cells and the activation of this TA system (Goormaghtigh et al., 2018a). Moreover, we recently showed that another TA system, MqsrA (see below), that was thought to be a global regulator involved in stress responses and biofilm formation, does not appear to play any significant role neither in oxidative or bile stresses nor in macrocolony formation (Fraikin et al., 2019). Therefore, our data strongly argue against the idea that TA systems are pivotal elements of antibiotic persistence. This basically leaves the principal questions in the field open (for a recent review see Fraikin et al., 2020). Conditions in which TA systems are activated and what the outcomes of such activations are, still remain undetermined.

**FIGURE 1:**
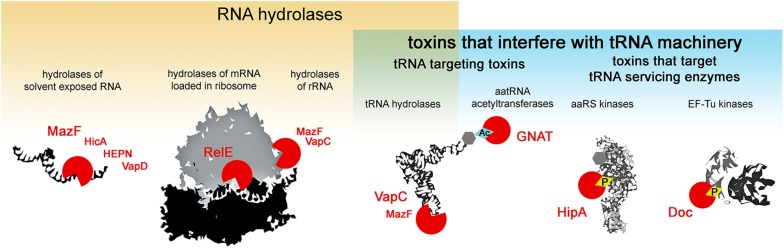
Activities of type II TA toxins. Cellular targets of the toxins are depicted in black and white; toxins are depicted as red circles – open circles for toxins hydrolyzing chemical bonds, circles with diamond for toxins transferring chemical groups on the targets. Ac stands for acetylation, and P for phosphorylation.

Type II toxins are very diverse in their molecular mode of action, however, almost all the families described to date comprise toxins that target protein synthesis (Harms et al., 2018). Several major mechanisms of translation inhibition by type II toxins can be distinguished: RNA hydrolysis of (i) solvent exposed RNAs, (ii) mRNAs in ribosomes, (iii) rRNAs, (iv) tRNAs or interference with the tRNA machinery, where in addition to the above mentioned hydrolysis of tRNAs, toxicity is exhibited by the modification of tRNA cargo (v) and the inactivation of enzymes that service the tRNAs (vi) i.e., phosphorylation of aminoacyl-tRNA synthetases that charge the tRNAs or EF-Tu which delivers the tRNAs to the ribosome (Figure 1). Most type II toxin activities lead to the general inhibition of protein synthesis and the subsequent inhibition of growth. In this review, we follow this classification to discuss the specificity and evolution of the different families of toxins from TA systems. We discuss the consequences of toxin-mediated translation inhibition on cell physiology and phenotypes and raise questions about their biological functions in light of recent discoveries.

## Hydrolysis of Rnas

### MazF Toxins

**FIGURE 2:**
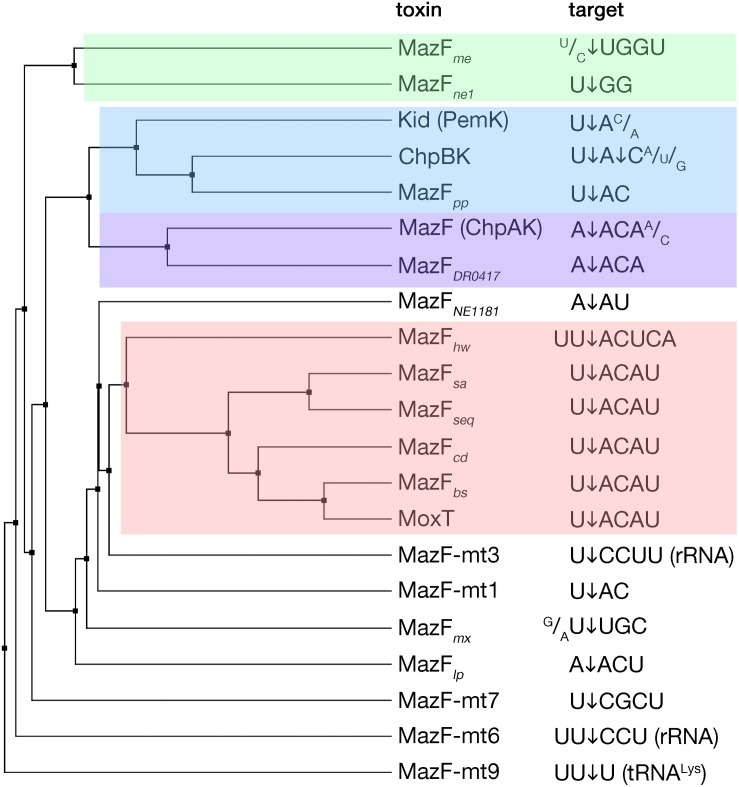
MazF toxins and their specificity of RNA cleavage. MazF protein sequences were aligned and an average distance tree was build (BLOSUM 62, JalView) (Waterhouse et al., 2009). Substrate specificity is indicated (right). The cleavage position of the substrate is indicated by an arrow. Subsets of toxins sharing similar target sequences are boxed with different colors. Protein identifiers and cleavage specificity were taken from: *E. coli* MazF (ChpAK) (NP_417262.1) (Culviner and Laub, 2018; Mets et al., 2019), ChpBK (NP_418646.1) (Zhang et al., 2005), Kid (PemK) (YP_003937673.1) (Munoz-Gomez et al., 2004; Zhang et al., 2004); *Bacillus subtilis* MazF*_*bs*_* (AOR96854.1) (Park et al., 2011); *Bacillus anthracis* MoxT (NP_842807.1) (Verma and Bhatnagar, 2014); *Staphylococcus aureus* MazF*_*sa*_* (BBJ19047.1) (Zhu et al., 2009); *Staphylococcus equorum* MazF*_*seq*_* (AFV93478.1) (Schuster et al., 2013); *Clostridium difficile* MazF*_*cd*_* (YP_001089981.1) (Rothenbacher et al., 2012); *Legionella pneumophila* MazF_*lp*_ (CCD10720.1) (Shaku et al., 2018); *Deinococcus radiodurans* MazF*_*DR0417*_* (AAF09995.1) (Miyamoto et al., 2017); *Haloquadratum walsbyi* MazF*_*hw*_* (WP_048066888.1) (Yamaguchi et al., 2012); *Pseudomonas putida* MazF*_*pp*_* (NP_742932.1) (Miyamoto et al., 2016a); *Myxococcus xanthus* MazF*_*mx*_* (SDX28280.1) (Nariya and Inouye, 2008); *Methanohalobium evestigatum* MazF*_*me*_* (WP_013195679.1); *Nitrosomonas europaea* MazF*_*ne1*_* (WP_011111532.1) (Miyamoto et al., 2018), MazF*_*NE1181*_* (CAD85092.1) (Miyamoto et al., 2016b); *Mycobacterium tuberculosis* MazF-mt1 (NP_217317.1) (Zhu et al., 2006), MazF-mt3 (NP_216507.1) (Zhu et al., 2008; Schifano et al., 2014), MazF-mt6 (NP_215618.1) (Schifano et al., 2013), MazF-mt7 (NP_216011.1) (Zhu et al., 2008), MazF-mt9 (YP_004837055.2) (Barth et al., 2019).

MazF toxins are RNA endonucleases that exhibit cleavage specificity to sequences spanning from three to seven bases (Figure 2). Most of the MazF toxins prefer U upstream of cleavage (at position −1) and AC downstream of cleavage (at positions +1 and +2, respectively) with less stringency outside of the two to four main recognized bases (Figure 2). The *E. coli* MazF cleaves in the coding as well as untranslated regions of mRNAs, independent from the reading frame, and in rRNA precursors (Mets et al., 2017; Culviner and Laub, 2018; Mets et al., 2019). Slight changes in sequence specificity of different MazF enzymes from different bacteria, correspond well to their amino acid sequence similarity (Figure 2). A particular member of the MazF family, the MazF-mt9 toxin from *Mycobacterium tuberculosis*, cleaves a tRNA substrate (Schifano et al., 2016), similar to the VapC toxins that will be discussed later in this review.

**FIGURE 3:**
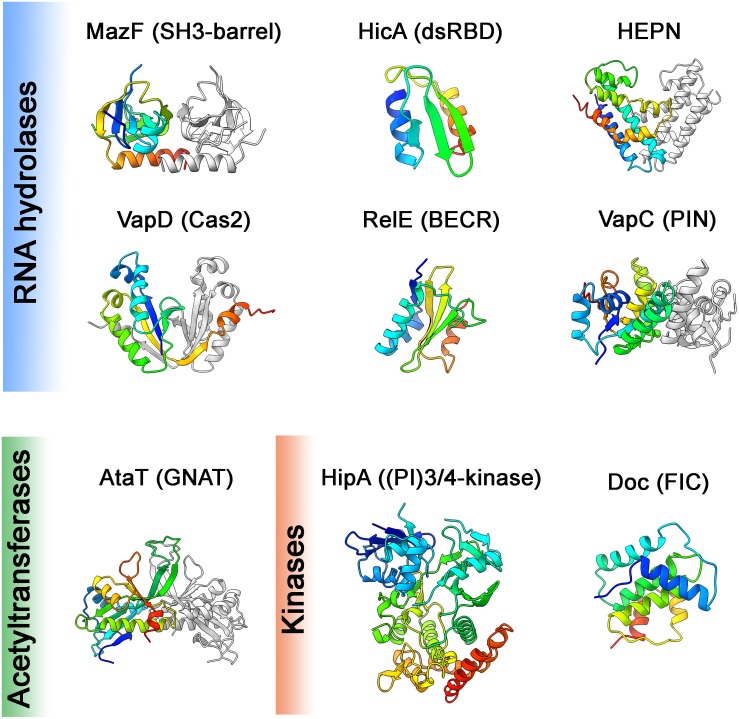
Toxin structures. Structures of the toxins from different families representing different structural folds (noted in brackets) are shown. These domains are common in proteins of the defense and offense systems (HEPN, Cas2, BECR, FIC), RNA processing (dsRBD, PIN), eukaryotic cell signaling (SH3-barrel, (PI)3/4-kinase) or various other functions (GNAT). Structures are colored in rainbow from N-terminus (blue) to C-terminus (red), in cases of dimers the second monomer is in gray. Structures were visualized using ChimeraX, PDB codes were following: *E. coli* MazF:3nfc; *E. coli* HicAB:6hpb (Manav et al., 2019); *S. oneidensis* SO_3166-SO3165 (HEPN): 5yep (Jia et al., 2018); *H. pylori* HP0315 (VapD): 3ui3 (Kwon et al., 2012); *S. flexneri* VapC^*D7A*^: 5ecw (Xu et al., 2016); *E. coli* RelE^*R81A, R83A*^: 2kc9 (Li et al., 2009); *E. coli* HipA^*S150A*^: 3tpb (Schumacher et al., 2012); *E. coli* AtaT^*Y144F*^: 6gtp (Jurenas et al., 2019); prophage P1 Phd-Doc: 3k33 (Garcia-Pino et al., 2010). In the cases of structures in complexes with antitoxin, the coordinates of antitoxin were deleted.

The structures of many MazF toxins alone or in complex with an mRNA substrate or with their cognate antitoxins have been solved (Hargreaves et al., 2002; Kamada et al., 2003; Simanshu et al., 2013; Zorzini et al., 2014, 2016; Ahn et al., 2017; Chen et al., 2017; Hoffer et al., 2017). The MazF monomer consists of 2 beta-sheets composed of antiparallel beta-strands linked by three or four small alpha-helices (Figure 3). The residues constituting the active site are located on the β1-β2 and β3-β4 linkers (Kamada et al., 2003; Simanshu et al., 2013; Zorzini et al., 2016). Although MazF toxins possess what is known as the SH3-barrel-fold, they are not related to other members of this fold (Anantharaman and Aravind, 2003). Two MazF subunits form a dimer with an extensive dimeric interface. A concave positively charged groove at the interface between the two subunits of the MazF dimer binds RNA in an extended alignment with bases facing upwards toward the groove (Simanshu et al., 2013; Zorzini et al., 2016). Structural studies have shown that a MazF dimer binds to one mRNA molecule and covers at least seven bases (Simanshu et al., 2013; Zorzini et al., 2016), explaining the extent of possible recognition and necessity of an unstructured RNA substrate, i.e., solvent exposed bases. MazF cleavage leaves 2′, 3′-cyclic phosphate at the 3′-end and 5′-OH group at the 5′-end of the cleavage site, which are used as a signature to study the cleavage products generated by the *in vivo* overexpression of MazF toxins (Schifano et al., 2014; Mets et al., 2017).

In addition to cleaving mRNAs, the *E. coli* MazF toxin was shown to cleave the 16S rRNA (Vesper et al., 2011). It was proposed that rRNA-cleavage generates specialized ribosomes able to translate specific pools of mRNAs constituting a regulon that is required to cope with various stresses (Vesper et al., 2011; Sauert et al., 2016). The model relies on data showing that *E. coli* MazF cleaves the 16S rRNA in the decoding center before ACA sequences at nucleotide positions 1396 and 1500 (↓^1396^ACA and ↓^1500^ACA) within the 30S ribosomal subunit. It was speculated that MazF-mediated cleavage removes the anti-SD sequence, thereby generating specialized ribosomes that are able to translate specialized MazF-processed leaderless mRNAs that also lack SD (Vesper et al., 2011; Sauert et al., 2016). However, these particular 16S rRNA bases are paired in the 30S subunits, making the MazF-dependent cleavage very unlikely. It was later shown that indeed, MazF cleaves the rRNAs in their precursor states, before the 30S subunit biogenesis, and at multiple sites (Mets et al., 2017; Culviner and Laub, 2018). This is in agreement with structural data showing that MazF binds to single-stranded RNA (ssRNA) and interacts with the bases (Simanshu et al., 2013; Zorzini et al., 2016). In fact, fragmentation of rRNAs as well as mRNAs including those coding for ribosomal proteins leads to the accumulation of aberrant ribosomal subunits and generates irregular particles with fragmented rRNA upon MazF expression (Mets et al., 2017). Accordingly, the synthesis of the vast majority of cellular proteins drop in response to MazF cleavage, without enrichment of any specific functional protein groups. Proteomic studies upon MazF expression did not find any ‘death’ or ‘survival’ proteins (Mets et al., 2019), associated to the proposed programmed cell death pathway (Amitai et al., 2009).

The TA-mediated programmed cell death (PCD) theory proposed that some TA systems, in particular MazEF, serve as built-in suicide modules (Engelberg-Kulka et al., 2004). Multiple different and unrelated stressing conditions, such as amino acids starvation and elevated ppGpp (guanosine tetraphosphate) concentrations, antibiotic treatments, high temperature, H_2_O_2_ treatment or phage infections were proposed to trigger MazF-dependent PCD. All these conditions would lead to the inhibition of *mazEF* expression. Since the MazE antitoxin is unstable and degraded by ATP-dependent proteases, this would liberate MazF and provoke the death of a large subpopulation of cells (Engelberg-Kulka et al., 2004). Within the frame of this pathway, it has been suggested that MazF itself is induced by a ‘quorum sensing’ peptide called extracellular death factor (EDF). This pentapeptide was identified to have the NNWNN sequence (Kolodkin-Gal et al., 2007) and is thought to amplify the endoribonucleolytic activity of MazF and other homologs and prevent the interaction of MazF toxins with their cognate antitoxins (Belitsky et al., 2011). However, alongside the PCD theory, the involvement of the EDF peptide in MazF-mediated PCD is questionable. First, in the original paper that describes the activity of the EDF peptide, the authors observed a drastic decrease in bacterial viability upon the addition of 2.5 ng/ml (4 nM) of EDF to growing *E. coli* cultures. This effect was also observed in MazF-deleted strains although at higher concentrations, starting from 200 ng/ml (300 nM) (Kolodkin-Gal et al., 2007). In subsequent publications, the authors showed that *in vitro* MazF ribonucleolytic activity was enhanced by only 26% by the addition of 1.5 μM of EDF (Belitsky et al., 2011). Further addition of up to 7.5 μM of EDF increased MazF activity to a maximum of 57% (Belitsky et al., 2011). Such a high EDF concentration, corresponding to a 300-fold molar excess, was used to show the disruption of the MazE-MazF complex *in vitro*. Assuming that EDF is produced *in vivo* under physiological conditions, it is very unlikely that such high concentrations will be reached. Moreover, *in vivo*, viability of bacteria is highly affected at EDF concentrations that are by several logs lower (4 nM versus 7.5 μM). The same group later discovered more EDF-like peptides (6 aa or longer) in *Bacillus subtilis* and *Pseudomonas aeruginosa*, however all of these peptides were only active in combination with rifampicin (Kolodkin-Gal et al., 2007; Kumar et al., 2013). Although in the original studies, rifampicin was used to induce MazF-mediated PCD (Kolodkin-Gal et al., 2007; Kumar et al., 2013), other studies did not find any MazF-related effect of rifampicin, nor any MazF-mediated PCD (Tsilibaris et al., 2007; Ramisetty et al., 2016). Thus, the cellular functions of MazF remain an open question.

Probably, the most intriguing case is that of *M. tuberculosis* which encodes 10 MazF toxins among its large TA arsenal (Sala et al., 2014). Out of those, the MazF-mt3 and MazF-mt6 toxins cleave mainly mRNAs at the U↓CCUU and UU↓CCU sequences, respectively. In addition, MazF-mt3 cleaves the anti-SD sequence of the 16S rRNA and both the MazF-mt3 and MazF-mt6 cleave the 23S rRNA loop 70 (L70) opening to the ribosomal A site (Schifano et al., 2013, 2014; Hoffer et al., 2017). It has been shown that MazF-mt6 can cleave the 23S rRNA in mature 50S subunit, albeit with 30% efficiency as compared to free RNA fragment coding for 23S L70 sequence (Hoffer et al., 2017). This indicates that like other MazF toxins, MazF-mt6 cleaves the solvent exposed target sequence. Alternatively, cleavage might be possible in rRNA precursors as described for *E. coli* MazF (Mets et al., 2017; Culviner and Laub, 2018). Another MazF toxin from *M. tuberculosis*, MazF-mt9, cleaves the tRNA^*Lys*43–UUU^ in its anticodon sequence (Schifano et al., 2016; Barth et al., 2019). Since tRNA^*Lys*19–*CUU*^ does not compensate for tRNA^*Lys*43–UUU^, ribosomes stall at the AAA codon in MazF-mt9 overexpression conditions (Barth et al., 2019). Ribosome stalled transcripts are further cleaved by specific RNAses such as RNAse J and are therefore eliminated (Barth et al., 2019). Since the AAA codon is rare in the GC-rich *M. tuberculosis* (5.3/1000), the authors speculate that expression of MazF-mt9 generates a specific proteome consisting of the proteins whose genes are poor in AAA Lys codons (Barth et al., 2019). However, it remains unclear whether genes devoid of the AAA codon would be sufficient to generate a functional proteome.

Interestingly, MazF toxins share high structural similarity to the F plasmid CcdB toxin (Loris et al., 1999; Hargreaves et al., 2002; Gogos et al., 2003; Kamada et al., 2003) that binds to the GyrA subunits of DNA-gyrase and induces double-strand breaks and the SOS response (Bernard and Couturier, 1992; Bernard et al., 1993; Dao-Thi et al., 2005). Despite their structural similarity, the two toxins bind their substrates using different sites on the toxin dimer interface – CcdB binds GyrA *via* the α4 helices, while, MazF recognizes RNA using the β1-β2, β3-β4, β4-β5 loops and a short α1 helix (Dao-Thi et al., 2005; Zorzini et al., 2016). On the other hand, the activity of MazF and CcdB toxins is regulated by their cognate antitoxins in a common way, which further supports the hypothesis of a common ancestor (Zorzini et al., 2016).

### HicA Toxins

HicA toxin and its cognate HicB antitoxin owe their gene names to a genetic locus linked to the pilus gene cluster (hif contiguous) in *Haemophilus influenzae* (Mhlanga-Mutangadura et al., 1998). HicA toxins possess small ∼50 amino acid double-stranded RNA binding domains (dsRBD). HicA folds into a three-stranded antiparallel beta-sheet flanked by two alpha helices that reside on one side of the sheet (Figure 3) (Makarova et al., 2006; Butt et al., 2014). The positively charged surface is predicted to bind RNA and the catalytic histidine residue located in the β2 strand is required for RNase activity (Bibi-Triki et al., 2014; Butt et al., 2014; Kim et al., 2018). The *E. coli* HicA toxin cleaves mRNAs and tmRNA *in vivo* independently of translation and no consensus of cleavage was reported (Jorgensen et al., 2009). *Yersinia pestis* HicA3 was shown to degrade *in vitro* transcribed mRNAs (Bibi-Triki et al., 2014) and *Sinorhizobium meliloti* HicA degrades purified rRNA (Thomet et al., 2019). However, there is not enough data available to be able to conclude what the precise targets of HicA toxins are *in vivo*.

Outside of the TA context, it has been shown that the dsRBD domain binds double-stranded RNA where the α1 helix interacts with the minor groove and the α2 helix with the major groove of RNA molecules (Ryter and Schultz, 1998). All the HicA toxins studied to date hydrolyze RNA in addition to binding (Jorgensen et al., 2009; Bibi-Triki et al., 2014; Thomet et al., 2019), however there are no details on the molecular substrate binding and hydrolysis.

The typical antitoxin partner HicB protein comprises DNA-binding domain fused to a degenerated RNAse H-fold (Makarova et al., 2006). These two domains (dsRBD and RNAse H) are also found in the architecture of eukaryotic RNA interference (RNAi) machinery (Makarova et al., 2006).

### HEPN-Fold Toxins

The HEPN (Higher Eukaryotes and Prokaryotes Nucleotide-binding domain) superfamily contains proteins that have all-alpha helical catalytic domains. Outside of the TA context, HEPN-domain proteins typically have metal-independent endonuclease activities, although some only bind RNA without degrading it (Anantharaman et al., 2013). HEPN domains are shared between TA and prokaryotic defense systems, such as abortive infection modules, restriction-modification systems and CRISPR-Cas systems, as well as eukaryotic antiviral, antitransposon systems and rRNA processing enzymes (Anantharaman et al., 2013). In the TA context, HEPN is frequently found in association to MNT (minimal nucleotidyltransferase) domain proteins. *Shewanella oneidensis* SO_3166 toxin possesses a HEPN domain and its cognate antitoxin SO_3165 an MNT domain (Yao et al., 2015). The SO_3166 toxin cleaves mRNA, but not rRNA or tRNA *in vitro*, however, the sequence specificity has not been determined. The toxin has a typical all-alpha helical HEPN fold and conserved Arg-(4-6X)-His motif, and forms a dimer with a potential composite active site in a central cleft that could accommodate RNA substrate (Figure 3) (Jia et al., 2018).

RnlA (or RNase LS)-like toxins that exhibit RNAse activity (Otsuka et al., 2007) also contain a catalytic domain belonging to the HEPN superfamily (Anantharaman et al., 2013). RNA cleavage by RnlA was shown to be dependent on translation (Otsuka and Yonesaki, 2012), more specifically on translation termination (Yamanishi and Yonesaki, 2005). RnlA induces sequence non-specific mRNA cleavage more frequently occurring 3′ to pyrimidines (Kai and Yonesaki, 2002). Homologous toxin LsoA shares the same cleavage pattern, however, cleavage sites are not identical (Otsuka and Yonesaki, 2012). The RnlA and LsoA toxins were shown to be part of functional TA systems with their cognate antitoxins RnlB and LsoB, respectively (Koga et al., 2011; Otsuka and Yonesaki, 2012). Interestingly, it was shown that the RnlA and LsoA toxins have a common antitoxin, Dmd, encoded by the T4 phage (Otsuka and Yonesaki, 2012). This antitoxin does not share any sequence similarity with the homologous but not interchangeable RnlB and LsoB antitoxins. T4 phages devoid of the Dmd-encoding gene are unable to propagate on *E. coli*, opening the possibility that the RnlAB and LsoAB systems act as defense mechanisms (Kai and Yonesaki, 2002; Otsuka and Yonesaki, 2012). Later it was shown that the activity of the RnlA and LsoA toxins is enhanced by RNAse HI (Naka et al., 2014) – an RNase responsible for RNA cleavage in DNA-RNA duplexes and removing the RNA primer in DNA replication (Miller et al., 1973; Itoh and Tomizawa, 1980). RNase H-fold domains are often fused to HEPN domains in larger architectures, suggesting that other RNase LS family proteins could also target RNA in DNA–RNA duplexes (Anantharaman et al., 2013). RnlA toxins in addition to the HEPN domain (also described as DBD) contain two additional domains, NTD and NRD that share similar topology of 4–5 stranded antiparallel beta-sheets with two alpha helices. It was shown that the DBD domain is responsible for dimerization of the toxin, its toxicity, and it is neutralized by both the RnlB and Dmd antitoxins (Wei et al., 2013). The functions of the NTD and NRD domains still need to be investigated.

### Cas2-Like VapD Toxins

The *vapD* gene was first detected in the chromosome of the anaerobic bacterium *Dichelobacter nodosus*. The genes of this locus, prevalent in virulent strains, were designated as *vapABCD* (Katz et al., 1992). The *vapBC* locus was later shown to encode a type II TA system (Daines et al., 2007); the same was demonstrated for *vapD* and a small upstream encoded ORF designated *vapX* (Daines et al., 2004). Later it was found that the VapD toxin from *Helicobacter pylori* displays a ferredoxin-like fold and therefore is structurally related to CRISPR-associated protein Cas2 (Figure 3). VapD functions as an endoribonuclease and cleaves mRNA preferentially before A or G nucleotides (Kwon et al., 2012). The presence of solitary *vapD* toxin in the *Neisseria gonorrhoeae* pEP5289 plasmid was suggested to be a factor restricting plasmids’ host range. A small cryptic neisserial plasmid pJD1, however, contains full VapXD. When the VapX antitoxin from pJD1 is expressed in *E. coli*, it increases the conjugation rate of pEP5289. This suggests that solitary VapD could limit the host range of pEP5289-like plasmids to the ones that contain the pJD1 plasmid carrying an intact VapXD module, and is therefore able to neutralize incoming VapD (Pachulec and van der Does, 2010). Although it has been proposed that *vapXD* locus as well as *vapBC* locus contributes to the virulence of *H. influenzae* (Ren et al., 2012), no molecular mechanism has been demonstrated yet. VapXD satisfies the definition of the type II TA module and comprises the toxin that likely shares its origins with the Cas2 protein, however, virtually nothing else is known about its molecular mechanism or its physiological function.

## mRNA Cleavage in the Ribosome by RelE Toxins

Almost all RelE toxins cleave mRNAs in the A site of the ribosome, between the second and third position of the codon. RelE family toxins described to date share as low as 11–20% sequence identity but retain the conserved fold which is similar to ribosome independent endoribonucleases T1, Sa2, and U2 (Neubauer et al., 2009; Schureck et al., 2015). The well-studied RelE-like toxins (*E. coli* RelE, YoeB, YafQ, and *Proteus vulgaris* HigB) possess different active sites, have different preferences for targeted mRNA codons, and differ in their ability to associate with 30S and/or 70S ribosomes (Neubauer et al., 2009; Feng et al., 2013; Maehigashi et al., 2015; Schureck et al., 2016b; Pavelich et al., 2019). Analysis of the cleavage specificity of RelE homologs from a wide range of bacterial species or isolates shows that the cleavage specificity is not strict (Goeders et al., 2013). The specificity likely originates from subtle differences in the association with the ribosome, rather than recognition of specific mRNA bases. RelE toxins belong to the large superfamily of BECR-fold proteins (barnase-EndoU-colicin E5/D-RelE fold) that is found in different polymorphic toxin systems (Zhang et al., 2012).

RelE contains an antiparallel beta-sheet (usually 4-stranded) flanked by two to four surface-exposed alpha helices enriched with positively charged residues (Figure 3) (Neubauer et al., 2009; Schureck et al., 2016b). The positively charged residues that decorate the alpha helices and mediate interaction with the negatively charged 16S rRNA backbone are thought to be a unique feature of the ribosome-dependent RelE family of endoribonucleases (Maehigashi et al., 2015; Schureck et al., 2016b). Despite the overall structural similarity, the residues that comprise the active sites of several well-studied RelE toxins are different, as well as the ribosome conformation induced by these toxins (Neubauer et al., 2009; Feng et al., 2013; Maehigashi et al., 2015; Schureck et al., 2016b). Inside the ribosomal A site, RelE toxins reorient and activate the mRNA for 2′-OH-induced hydrolysis. Although the ribosome is not directly involved in catalysis, it is required to achieve the correct orientation of the mRNA for the cleavage reaction (Neubauer et al., 2009). Generally, RelE toxins induce strong reorganization of the mRNAs at the ribosomal A site and cause the hydrolysis between the second and third position of the codon in the A site (Neubauer et al., 2009; Feng et al., 2013; Goeders et al., 2013; Maehigashi et al., 2015; Schureck et al., 2016b). Toxins pull the mRNA out of its typical tRNA bound state. In presence of the toxin, all three A site nucleotides are shifted by more than 7 Å (Neubauer et al., 2009; Schureck et al., 2016b). Conserved residues of the toxin orient the first two A site codon bases for hydrolysis, while the third base is usually oriented with the aid of 16s rRNA (Neubauer et al., 2009; Schureck et al., 2016b). Different RelE family toxins interact with all three nucleotides of the cleaved codon in different ways, leading to subtle specificities for nucleobases at each position (Neubauer et al., 2009; Feng et al., 2013; Maehigashi et al., 2015; Schureck et al., 2016b).

The *E. coli* RelE toxin extensively relies on the ribosome for both mRNA binding and cleavage, as it has lost conserved histidine and glutamate residues used for RNA cleavage in ribosome independent RNases like RNAse T1 (Heinemann and Saenger, 1983; Takagi et al., 2005; Neubauer et al., 2009). RelE instead uses conserved basic residues both for interaction and catalysis (Neubauer et al., 2009). Almost 1/5 of the residues of RelE are basic and provide a large potential for an interaction with negatively charged RNA. In particular, a large accumulation of these residues is observed on the three helices that interact with the 16S rRNA. Other RelE toxins, such as HigB, YoeB, or YafQ do not rely on basic residues and instead function more like the RNase T1, through conserved histidine and glutamate (or histidine or tyrosine) residues for catalysis (Kamada and Hanaoka, 2005; Maehigashi et al., 2015; Schureck et al., 2016b). In addition, the RelE toxin leaves 2′-3′-cyclic phosphate at the new 3′ end (Neubauer et al., 2009), while others (YefM, YoeB, HigB) further hydrolyze it to a 3′-phosphate product (Feng et al., 2013; Maehigashi et al., 2015; Schureck et al., 2016b), like RNase T1 (Heinemann and Saenger, 1983).

Most of the RelE toxins display virtually no codon specificity and cleave between the second and third positions of the codon with the only conserved preference of purines at the third position (Goeders et al., 2013). However, several RelE family toxins were reported to be selective for the AAA lysine codon. This codon is preferred by the *E. coli* YafQ, *Rhodopseudomonas palustris* RelE_*Rpa*_, *Nostoc* sp. RelE_*Nsp*_, *Sinorhizobium meliloti* RelE_*Sme*_ and *Treponema denticola* RelE_*Tde*_ (Prysak et al., 2009; Goeders et al., 2013). *P. vulgaris* HigB cleaves A-rich codons (Hurley and Woychik, 2009; Schureck et al., 2016b), and was shown to cleave the 30S bound mRNA, indicating that it can interfere with the initiation step of translation after IF1 dissociation (Schureck et al., 2016a). The AAA and other A-rich codons are the most frequent at the beginning of the ORFs in *E. coli* (Sato et al., 2001). Several studies have shown that the AT-rich content at the 5′ of the ORF likely reduces the secondary structures and has been shown to serve as a translation ramp for efficient protein expression in *E. coli* (Goodman et al., 2013; Verma et al., 2019). Moreover, ribosome profiling indicates that ribosomes spend a lot of time at the beginning of the transcripts (Oh et al., 2011), which might provide more time for these toxins to access their substrates. Therefore, at least a subset of RelE toxins could be considered as translation initiation inhibitors.

Several RelE toxins were shown to cleave at different sites with respect to the codon. For example, *E. coli* YhaV and *Mycobacterium avium* RelE cleave mRNA preferably between the codons (i.e., after the third base of the codon) (Goeders et al., 2013; Choi et al., 2017). However, the molecular mechanisms of substrate recognition are not yet described for these toxins. The YafO toxin was shown to be a ribosome-dependent endoribonuclease that cleaves mRNAs 11–13 bases downstream of the initiation codon (Zhang et al., 2009; Christensen-Dalsgaard et al., 2010). Such cleavage event was speculated to be located near the mRNA entrance tunnel rather than at the A site as determined for the RelE-like toxins (Schureck et al., 2016a). Although initially YafO was considered a RelE-like toxin (Christensen-Dalsgaard et al., 2010), the lack of structural information about this toxin or its interaction with ribosome lead to YafO being classified as a separate family (Leplae et al., 2011). Several RelE-family toxins, such as *E. coli* MqsR (YgiU), *Brucella abortus* BrnT and *H. pylori* HP0894 are suggested to cleave RNA in a ribosome-independent manner (Brown et al., 2009; Christensen-Dalsgaard et al., 2010; Han et al., 2011; Heaton et al., 2012) and could therefore be functionally closest to ribosome independent RNases T1, Sa2, and U2 found outside of the TA context. MqsR is a RelE-fold toxin that possesses an additional beta strand (Brown et al., 2009; Christensen-Dalsgaard et al., 2010). MqsR preferentially cleaves the 5′-G↓CN-3′ triplet (where N is preferentially U, but C or A are tolerated) in mRNA or rRNA precursors (Yamaguchi et al., 2009; Mets et al., 2017). The *H. pylori* HP0894 toxin prefers purines upstream of cleavage and its major cleavage activity was observed between first and second base at termination codons UAA and UAG (Han et al., 2011), therefore its independence from the ribosome is questionable. In fact, *E. coli* RelE was also suggested to inhibit translation termination, as in addition to CAG sense codon, it cleaves UAG or UAA stop codons between the second and third nucleotide and subsequently prevents class 1 release factors from binding the ribosome (Pedersen et al., 2003). It remains unclear whether RelE toxins targeting translation initiation would be able to compete for the A site with initiation factors, and those acting during elongation with tRNA. To date, only the affinity of YafQ toxin to the 70S assembled complex has been reported (∼360 nM) and is comparable to those of the above-mentioned factors (Maehigashi et al., 2015). It remains unclear why toxins rely on the ribosome for activity as simply blocking the translation cleavage of free mRNA would both be sufficient and efficient. It has been suggested that this dependence may indicate a specialized mechanism that would allow a response to stresses (Schureck et al., 2016a). On the other hand, selectivity for specific ribosomal complexes, such as initiating ribosomes, might be an efficient way to inhibit translation and is likely to give the same effect of reducing the global translation rate as ribosome-independent but more sequence-specific mRNA cleavage.

Bioinformatic searches and structural comparisons have detected the relationship between RelE-family toxins and those similar to ParE encoded by RK2 plasmid (Anantharaman and Aravind, 2003; Sterckx et al., 2016). ParE-family toxins act at the level of DNA replication by poisoning DNA-gyrase or by currently unidentified mechanisms (Jiang et al., 2002; Hallez et al., 2010; Yuan et al., 2010; Sterckx et al., 2016). Some residues are highly conserved across the RelE/ParE superfamily (Anantharaman and Aravind, 2003) and their three-dimensional structure is strikingly similar (Dalton and Crosson, 2010; Sterckx et al., 2016). The major differences between the mRNAses and replication inhibitors are the extended N-terminal alpha helices as well as the absence of the C-terminal helix observed in ParE toxins (Dalton and Crosson, 2010; Sterckx et al., 2016). ParE toxins are also devoid of the major catalytic residues used by RelE for mRNA cleavage in the ribosome (Neubauer et al., 2009; Dalton and Crosson, 2010). Evidence of the evolutionary relationships between RelE-like and ParE-like toxins, is the conserved principle of the binding of their cognate antitoxins to conserved hydrophobic motifs on the toxins, although different families of antitoxins can associate with both ParE and RelE-like toxins (Dalton and Crosson, 2010; Leplae et al., 2011; Sterckx et al., 2016).

## Interference With tRna Functions

### VapC Toxins

**FIGURE 4:**
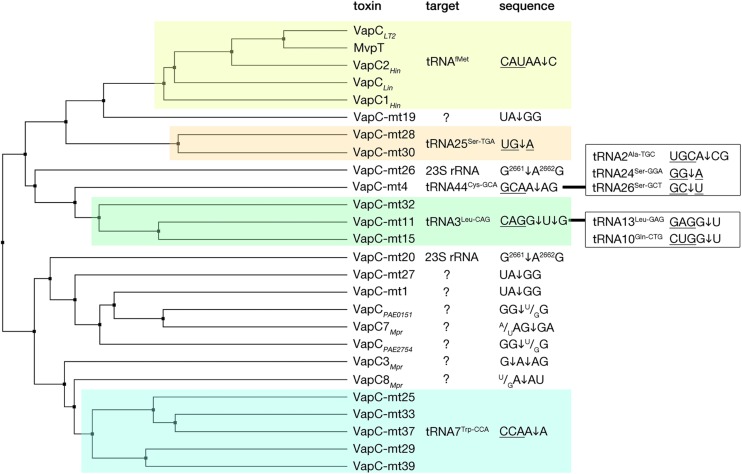
VapC toxins and their specificity of RNA cleavage. VapC protein sequences were aligned and an average distance tree was build (BLOSUM 62, JalView) (Waterhouse et al., 2009). Substrate specificity is indicated (right). Toxin cleavage position is marked by an arrow, tRNA anticodon sequence is underlined, in the case of rRNA numbers of nucleotide positions are indicated in superscript. Additional targets and sequences are marked in the boxes on the right. Subsets of toxins sharing similar target sequences are boxed with different colors. Protein identifiers and cleavage specificity were taken from: *Haemophilus influenzae* VapC1*_*Hin*_* (NP_438487.1), VapC2*_*Hin*_* (NP_439108.1) (Walling and Butler, 2018); *Salmonella enterica* VapC*_*LT2*_* (NP_461950.1), *Shigella flexneri* MvpT (AMN61047.1) (Winther and Gerdes, 2011); *Leptospira interrogans* VapC*_*Lin*_* (AAS71220.1) (Lopes et al., 2014); *Mycobacterium tuberculosis* VapC-mt4 (NP_215109.1) (Cruz et al., 2015; Winther et al., 2016), VapC-mt11 (NP_216077.1) (Winther et al., 2016; Cintron et al., 2019), VapC-mt15(NP_216526.1), VapC-mt25 (NP_214791.1), VapC-Mt28 (NP_215123.1), VapC-mt29 (NP_215131.1), VapC-mt30(NP_215138.1), VapC-mt32(NP_215630.1), VapC-mt33 (NP_215758.1), VapC-mt37 (NP_216619.1), VapC-mt39 (NP_217046.1) (Winther et al., 2016), VapC-mt20 (NP_217065.1), VapC-mt26 (NP_215096.1) (Winther et al., 2013, 2016), VapC-mt1 (NP_214579.1) (McKenzie et al., 2012; Sharrock et al., 2018), VapC-mt19 (NP_217064.1), VapC-mt27 (NP_215112.1) (Sharrock et al., 2018), *Pyrobaculum aerophilum* VapC*_*PAE2754*_* (WP_011008882.1), VapC*_*PAE0151*_* (WP_011007068.1) (McKenzie et al., 2012); *Metallosphaera prunae* VapC3*_*Mpr*_* (WP_012020824.1), VapC7*_*Mpr*_* (WP_012021162.1), VapC8*_*Mpr*_* (WP_012021192.1) (Mukherjee et al., 2017).

The VapC toxins are characterized by the presence of a PIN domain that presents a structural similarity with the classical Rossmann-fold associated with the binding of nucleotides and nucleotide-based cofactors (Rao and Rossmann, 1973; Matelska et al., 2017; Senissar et al., 2017). The PIN domain, although originally owing its name to the type IV pili protein PilT (PilT N-terminal like nucleases), is generally found in proteins that present various endonuclease functions such as tRNA and rRNA maturation, nonsense mediated mRNA decay, and DNA replication and repair in all domains of life (Matelska et al., 2017; Senissar et al., 2017). In the PIN motif, alternating beta strands and alpha helices (α/β/α sandwich) fold into a central five stranded parallel beta-sheet decorated with alpha helices on both sides (Figure 3). All VapC toxins, although presenting low sequence similarity, share conserved acidic residues (D, E, D, D/N) that are distant in the primary amino acid sequence, but cluster together in the protein to form an active site that coordinates divalent-cations, such as Mg^2+^ and Mn^2+^ that are required for cleavage of single-stranded RNA (Fatica et al., 2004; Daines et al., 2007; Das et al., 2014; Matelska et al., 2017; Senissar et al., 2017). VapC toxins cleave the 3′-O-P bond of single stranded RNA to produce 3′-hydroxyl and 5′-phosphate cleavage products (McKenzie et al., 2012). So far, VapC toxins targeting the initiation tRNA^*fMet*^, different elongation tRNAs, and the sarcin-ricin loop (SRL) of the 23 S rRNA have been identified (Figure 4) (Winther and Gerdes, 2011; Winther et al., 2013, 2016). All the VapC toxins targeting tRNAs cleave in the anticodon stem-loop (ASL), either in the anticodon sequence itself or at the 3′-side of the anticodon before the stem structure (Figure 4). The *M. tuberculosis* VapC-mt20 cleaves the 23S rRNA SRL loop in the helix 95 that structurally mimics the ASL of tRNAs (Winther et al., 2013), therefore all VapC toxins tested so far cleave ASL-like structures. Unlike other ribonuclease toxins (namely MazF and RelE – see above), VapC toxins appear to recognize both RNA sequence and structure (Winther et al., 2013; Cruz et al., 2015; Walling and Butler, 2018; Cintron et al., 2019). Ribonucleotide modifications in the ASL region might be important for governing the specificity of VapC toxins (Winther et al., 2016; Cintron et al., 2019). Strikingly, VapC-mt20 requires the presence of the ribosome for 23S rRNA cleavage and does not cleave isolated SRL rRNA fragments (Winther et al., 2013). Since the helix 95 is exposed to the solvent at the surface of the ribosome (Yusupov et al., 2001), it is accessible for cleavage, however no information of VapC-mt20 interactions with ribosomes is currently available (Winther et al., 2013).

While some VapC toxins cleave tRNAs that service highly abundant codons, others cleave tRNAs that service rare codons, however it is unlikely that they could be compensated by other tRNAs (Cruz et al., 2015; Winther et al., 2016; Cintron et al., 2019). Therefore, it is most likely that the overexpression of VapC toxins leads to global inhibition of translation. Breaks in the ASL will interfere with the aminoacylation of those tRNAs that are recognized through their ASL by aminoacyl-tRNA synthetases (McClain et al., 1998), while charged tRNAs cleaved in ASL are unlikely to generate productive interaction with the A site of the ribosome. It has been proposed that tRNA halves produced by VapC toxins could have further functions in the stress response (Cruz et al., 2015), however the lifetime and possible interactions of VapC cleavage products remain unexplored.

Cleavage of tRNA^*fMet*^ reported for *Salmonella*, *Shigella*, and several other VapC toxins (Winther and Gerdes, 2011) (Figure 4) will lead to inhibition of the initiation – a rate limiting step of protein synthesis (Laursen et al., 2005). It has been shown that expression of VapC_*LT*__2_, which leads to cleavage of initiator tRNA^*fMet*^, would boost the translation start from elongation codons (Winther and Gerdes, 2011). However, translation of only one model mRNA was followed (Winther and Gerdes, 2011), therefore it is difficult to conclude whether induction of VapC would lead to synthesis of specific proteins initiating with other codons and whether it would lead to a sufficient, functional, and specialized proteome.

Several VapC toxins were reported to cleave the SRL loop of 23S rRNA (Winther et al., 2013; Winther et al., 2016) (Figure 4) that owes its name to two toxins – the α-sarcin that cleaves it at the identical position as VapC-mt20 and the ricin that removes the adenine base in the SRL (Olmo et al., 2001; Grela et al., 2019). The SRL loop is located in the GTPase associated center of the large ribosomal subunit where translational GTPases are recruited. The SRL loop is required for docking and stimulation of GTP hydrolysis of all translation GTPases (Munishkin and Wool, 1997; Rodnina et al., 1999), therefore its cleavage leads to overall inhibition of protein synthesis in prokaryotes as demonstrated for VapC, as well as in eukaryotes in case of α-sarcin (Olmo et al., 2001; Winther et al., 2013).

Numerous structures of VapCs show that these toxins are dimers and that their cognate antitoxins neutralize the toxic activity by interfering with the binding of the metal ions (Miallau et al., 2009; Dienemann et al., 2011; Min et al., 2012). However, there is still no structural information regarding their interaction with the RNA substrates, making it difficult to predict details of target recognition and catalysis. It has been demonstrated that by comparing divergent VapCs that recognize particular tRNA or rRNA substrates, they can be grouped in sub-families sharing amino acid sequence similarity (Figure 4). Predicted specificity of new VapCs within these families was confirmed (Winther et al., 2016). Interestingly, VapC toxins can be encoded abundantly in one genome, with an outstanding example of *M. tuberculosis* encoding as much as 50 VapC toxins (Ramage et al., 2009; Sala et al., 2014). Despite the high abundance and sequence/structure similarities, these TAs appear to not cross-talk, at least for those that are functional and that have been tested – their toxins are neutralized only by their cognate antitoxins – although in some cases this insulation could be alleviated by a single mutation (Ramage et al., 2009; Walling and Butler, 2018). Moreover, mycobacterial VapC toxins do not overlap functionally, since many of them target different tRNAs or the SRL of 23S rRNA (Figure 4) (Winther et al., 2016). Nevertheless, the question of the functional benefit, if any, of having large arrays of VapCs, remains open.

### GNAT Toxins

GNAT-fold acetyltransferase toxins identified so far, including TacT, AtaT, and ItaT, acetylate aminocylated-tRNAs on the amino group of the cargo amino acids (Cheverton et al., 2016; Jurenas et al., 2017a; Wilcox et al., 2018). For convenience, we will refer to this family as AtaTs for Aminoacyl-tRNA-acetylating Toxins. GNAT-fold comprises a central beta-sheet composed of six to seven strands surrounded by four alpha helices (Figure 3). The alpha helix α3 located between the β4 and β5 strands encodes the signature motif R/Q-X-X-G-X-A/G, also referred to as ‘P-loop,’ which binds the pyrophosphates of acetyl-Coenzyme A which is used as a substrate for the transfer of the acetyl group (Neuwald and Landsman, 1997). The GNAT (general control non-repressible 5 (GCN5)-related *N*-acetyltransferases) family of proteins comprises more than 300,000 enzymes that acetylate various substrates from small metabolites to proteins and tRNAs (Ikeuchi et al., 2008; Salah Ud-Din et al., 2016). The GNAT-fold toxins from type II TA systems described to date form a distinct monophyletic group of GNAT acetyltransferases (Wilcox et al., 2018). Despite being related, these toxins have diverged to target different species of tRNAs charged with their respective amino acids (Jurenas et al., 2017b; Wilcox et al., 2018). The AtaT toxin encoded by the *E. coli* O157:H7 strain specifically acetylates the initiator tRNA Met-tRNA^*fMet*^. Acetylation of the Met loaded on the Met-tRNA^*fMet*^ impairs its interaction with the initiation factor 2 (IF2) and precludes the formation of the 30S translation initiation complex (Jurenas et al., 2017a). Formylation of the initiator fMet-tRNA^*fMet*^ is essential for normal growth (Shah et al., 2019) and is impaired by the Met acetylation, resulting in a strong growth inhibition and generating a dead-end product acMet-tRNA^*fMet*^. *In vivo* expression of AtaT manifests in accumulation of ribosome assembly intermediates, reflecting a strong inhibition of translation initiation (Jurenas et al., 2017a). Interestingly, AtaT is able to discriminate between the initiator Met-tRNA^*fMet*^ and the elongator Met-tRNA^*Met*^ *in vitro* (Jurenas et al., 2017a) although the molecular basis of this specificity has not yet been determined. The *E. coli* HS strain encoded-ItaT toxin acetylates the elongator Ile-tRNA^*Ile*^. This leads to the inhibition of translation elongation at Ile codons. It has been shown that tRNAs charged with N-blocked amino acid cannot form ternary complex EF-Tu:GTP:tRNA (Janiak et al., 1990), therefore acetylated elongator tRNAs would not be delivered to the ribosome. TacTs from *Salmonella* have been shown to have more relaxed specificities and target several elongator tRNAs. However, some specificity still occurs as different TacTs have slightly different preferences for subsets of elongation aa-tRNAs. For example, the TacT and TacT2 toxins mostly target the Gly-tRNA^*Gly*^, while TacT3 prefers Ile or Leu charged tRNAs (Rycroft et al., 2018). TacTs were suggested to play an important role for persistence of *Salmonella* in macrophages (Helaine et al., 2014; Cheverton et al., 2016; Rycroft et al., 2018). However, recent data did not find any involvement of TacT or other type II TA systems from *Salmonella* in persistence (Claudi et al., 2014; Pontes and Groisman, 2019). On the other hand, like other TAs, many AtaRT-like TA systems are associated to mobile genetic elements, such as plasmids, transposons and integrons and could be involved in their maintenance (Iqbal et al., 2015; McVicker and Tang, 2016; Jurenas et al., 2017b).

### HipA Toxins

HipA toxins phosphorylate aminoacyl-tRNA synthetases on conserved serines located in their ATP-binding sites, therefore leading to their inactivation (Kaspy et al., 2013; Vang Nielsen et al., 2019). Since phosphorylated aa-tRNA-synthetases cannot charge their respective tRNAs, ribosomes stall at the generated hungry codons. Consequently, an increase of ppGpp concentration is observed, associated to the activation of RelA, the effect known as the stringent response (Wendrich et al., 2002; Germain et al., 2013; Vang Nielsen et al., 2019). HipA toxins are serine-threonine kinases that belong to the phosphatidylinositol (PI) 3/4–kinase superfamily (Correia et al., 2006). Their C-terminal domain is all-alpha helical and has a similar fold to human CDK2/cyclin A kinase (Figure 3) (Schumacher et al., 2009). The C-terminal domain comprises the active site with a conserved aspartate, an ATP-binding ‘P-loop motif’ and a Mg^2+^ binding aspartate (Correia et al., 2006). The N-terminus is an α/β globular domain specific to the HipA toxins (Figure 3) (Correia et al., 2006; Schumacher et al., 2009). Interestingly, it can be encoded as a separate protein, as seen in a three-component TA system HipBST from *E. coli* O27. The HipT toxin exhibits sequence similarity with the C-terminal kinase region of the HipA toxin and also targets specific tRNA synthetase (see below). The gene located upstream of the *hipT* gene, *hipS*, encodes a small protein (∼100 amino acids) corresponding to the N-terminal domain of HipA which is able to counteract the toxic activity of HipT (Vang Nielsen et al., 2019). The first gene of the operon, *hipB*, encodes a protein that enhances the ability of HipS to counteract HipT. Indeed, it has been proposed, that in addition to HipB antitoxin, the N-terminal domain of HipA (similar to the HipS protein) could be involved in regulation of HipA toxin activity through dimerization that blocks the active site (Schumacher et al., 2015).

The *E. coli* K12 HipA toxin phosphorylates the Ser 239 located in the ATP-binding site of the glutamyl-tRNA synthetase (GltX) (Germain et al., 2013; Kaspy et al., 2013). The homologous *E. coli* O127 HipT toxin phosphorylates tryptophanyl-tRNA synthetase (TrpS) at the conserved Ser 197 (corresponding to Ser 239 in the glutamyl-tRNA synthetase) (Vang Nielsen et al., 2019). The Ser 239 of GltX is located in a conserved flexible loop (characteristic to type I aa-tRNA-synthetases). Conformational changes upon tRNA^*Glu*^ binding make this loop more exposed (Sekine et al., 2003). It was shown that HipA only phosphorylates the tRNA^*Glu*^-bound HipA (Germain et al., 2013). The conserved motif of GltX that is phosphorylated by HipA is required for ATP binding. Thus, phosphorylation of GltX likely precludes the binding of ATP at the first step of aminoacylation reaction (Sekine et al., 2003; Germain et al., 2013). Meanwhile, the HipT toxin is able to phosphorylate the TrpS tryptophanyl-tRNA synthetase independently of tRNA^*Trp*^ binding (Vang Nielsen et al., 2019). In fact, GltX activates glutamate to glutamyl adenylate only in presence of cognate tRNA, while TrpS can activate tryptophan to tryptophanyl-adenylate without binding tRNA^*Trp*^ (Giege and Springer, 2016). These differences likely correspond to the conformation and accessibility of the loop in the ATP-binding site of the targeted aminoacyl-tRNA synthetases.

It has been shown that HipA inactivates itself by auto-phosphorylation (Correia et al., 2006). Typically, kinases auto-phosphorylate on the solvent exposed activation loops, HipA instead auto-phosphorylates at Ser150 located in its ATP-binding site (P-loop motif) located in the core of the protein (Schumacher et al., 2012). Autophosphorylation on Ser150 leads to conformational changes of the P-loop motif which then hinders binding of ATP (Correia et al., 2006; Schumacher et al., 2012). Likely due to the flexibility of the P-loop, autophosphorylation is an intermolecular event and therefore is likely to happen when amounts of free HipA increase, thereby providing an auto-regulation and reported as a possibility to revive from HipA-induced growth inhibition (Korch and Hill, 2006; Schumacher et al., 2012). HipT also auto-phosphorylates at Ser57 and Ser59, which are adjacent to the P-loop motif of the kinase, and which corresponds to the position of autophosphorylation of HipA (Vang Nielsen et al., 2019). The HipA toxin from *Shewanella oneidensis* was also shown to auto-phosphorylate at a similar position and it was proposed that this modification is important for complex formation with the cognate HipB antitoxin and its further binding to DNA as well as the stability of this complex (Wen et al., 2014). The autophosphorylation most likely regulates the activity and the expression of the HipA toxins.

The discovery and the name of the *E. coli K12* HipA toxin is related to the isolation of the hyper-the HipA7 persistent mutant (Moyed and Bertrand, 1983). The HipA7 strain shows a 100 to 1000-fold increase in persistence (Korch et al., 2003). It was shown that the *hipA7* allele codes for the mutations G22S and D291A in the HipA protein, which was later described to encode the toxin HipA from the *hipBA* TA system (Black et al., 1991; Korch et al., 2003). Recently, a proteomics study suggested that HipA phosphorylates multiple targets in addition to the principal target GltX, notably the ribosomal protein L11 (RplK) and other proteins involved in translation, transcription, and replication (Semanjski et al., 2018). This is in agreement with previous reports showing that HipA overexpression inhibits protein, RNA and DNA synthesis *in vivo* (Korch and Hill, 2006). However, no phosphorylation resulting from endogenous HipA encoded on the chromosome was observed, since it is repressed by HipB (Semanjski et al., 2018). Furthermore, the HipA7 strain showed phosphorylation of GltX and to a lesser extent of the phage shock protein PspA (Semanjski et al., 2018). This is in agreement with previous observations that the G22S mutation in the N-terminal domain of HipA7 likely results in compromised dimerization and failure to form a HipA:HipB:operator-DNA complex required for neutralization of HipA and transcription repression of the *hipBA* locus (Schumacher et al., 2015). Induction of HipA7 however showed more phosphorylation targets (Semanjski et al., 2018), therefore indicating that they might be physiologically non-relevant and result from a high expression of the protein. Therefore, GltX is likely to be the main target of *E. coli* K-12 HipA toxin. HipA7 also showed less auto-phosphorylation than HipA, indicating that the mutant either has less activity or compromised folding or stability, as induction of chaperons was reported upon overexpression of HipA7 (Semanjski et al., 2018). Accordingly, it has been previously reported that the HipA7 does not have a strong inhibitory effect on protein synthesis (Korch and Hill, 2006). In conclusion, the *hipA7* allele likely results in smaller effective HipA7 concentrations as compared to the wild-type system and in overall reduced activity of phosphorylation, however the HipA7 toxin is more likely to be released from the HipBA7 complex. It was later proposed that the HipBA system, together with 10 other TA systems in which the toxins are RNases, are the central effectors of antibiotic persistence in *E. coli* (Maisonneuve et al., 2018a, b; Germain et al., 2019). However, it was later demonstrated that these TA systems are not involved in persistence (see above) (Ramisetty et al., 2016; Harms et al., 2017; Shan et al., 2017; Goormaghtigh et al., 2018a) and the idea that ppGpp is required for persistence has been challenged as well (Bhaskar et al., 2018; Pontes and Groisman, 2019).

Another family of proteins that are YjjJ-like belong to the same PI 3/4-kinase superfamily as HipA and possesses similarities in the catalytic domain (Correia et al., 2006; Maeda et al., 2017). YjjJ although encoded without cognate antitoxin was shown to be toxic, but strikingly it could be neutralized by the HipB antitoxin. YjjJ comprises a DNA-binding motif in its N-terminus that is not present in HipA-like toxins. YjjJ appears to have different cellular targets as it does not inhibit macromolecule synthesis and may affect cell division (Maeda et al., 2017).

### Fic/Doc Toxins

Doc toxin is a Fic-fold protein that catalyzes phosphorylation of the elongation factor EF-Tu (Castro-Roa et al., 2013). Fic-fold proteins typically perform NMPylation (AMPylation, GMPylation, or UMPylation) as a post-translational modification on proteins using a phosphate-containing compound, usually ATP (Garcia-Pino et al., 2014; Veyron et al., 2018). In contrast, Doc toxin catalyzes the transfer of the phosphor moiety of ATP, instead of transferring the AMP, and is therefore a kinase (Castro-Roa et al., 2013). Doc phosphorylates EF-Tu at the conserved threonine Thr283 which leads to translation arrest (Castro-Roa et al., 2013). The binding site of Doc on EF-Tu likely overlaps with the tRNA binding site since ternary complex formation prevents Doc binding. In agreement with that, Doc preferentially phosphorylates the GDP-bound state of EF-Tu (Castro-Roa et al., 2013). Phosphorylation of the Thr382 located on the loop of the beta-barrel domain III of EF-Tu locks it in an unfavorable open conformation typical of GDP-bound EF-Tu (Talavera et al., 2018). Conformational dynamics of EF-Tu are the essence of its function and GTP hydrolysis has a major effect on aa-tRNA binding and interaction with the ribosome. Once locked in an open state, EF-Tu exhibits decreased affinity for aa-tRNA to a similar extent as the affinity of GDP-bound EF-Tu (Talavera et al., 2018) and is not compatible with translation (Castro-Roa et al., 2013). Fic domain toxins that perform AMPylation have also been reported to constitute type II TA modules (Harms et al., 2015). FicT toxins target TopoIV and Gyrase, and block their ATPase activity (Harms et al., 2015). Fic and Doc domain families, together sometimes referred to as Fido proteins, have conserved the alpha-helical core arranged in the bundle with two additional alpha helices perpendicular to the bundle (Figure 3) (Kinch et al., 2009). Although active site geometry is conserved, a single substitution in the active site motifs for Doc in comparison to Fic (K73 in Doc while G114 in Fic) leads to inverted orientation of ATP and therefore the transfer of γ-phosphate (Castro-Roa et al., 2013).

In contrast to some TA toxins, like RnlA that targets invading phages, Doc is itself encoded by a phage and provides stability to its lysogenic state. The Doc toxin is encoded by the P1 phage that is maintained as a plasmid during its lysogenic cycle. Doc was named after its impact on the remarkable stability of lysogens due to elimination of cells that have lost P1 (death on curing) (Lehnherr et al., 1993).

## Conclusion and Discussion

### Translation as a Favorite Target

Protein synthesis is one of the most complex processes in the cell. Translation involves the step by step assembly of ribosomes, coordinated movements of translation machinery at every addition of a new amino acid into the nascent polypeptide chain and well-organized termination, leading to the release of a newly synthesized protein as well as the recycling of ribosomes and translation factors (Arenz and Wilson, 2016a). The complexity of this process provides a multitude of intervention possibilities that have been explored by antibiotics, bacteriocins and secreted toxins (Zhang et al., 2012; Arenz and Wilson, 2016b; Kumariya et al., 2019). Toxins that are part of type II TA systems target translation in a multitude of ways – from destroying the transcripts before or during translation, to affecting ribosome biogenesis or interrupting the charging of tRNAs or the delivery of amino acids into growing polypeptide chains. Targeting translation allows not only choices, but also room for specialization – potential targets include a great number of tRNAs, tRNA synthetases, translation factors, and the ribosome itself (rRNAs and ribosomal proteins). Specialization of toxins portrayed in this review is already seen in almost all the TA toxin families that we know of to date. MazF toxins have diverged to target different mRNA and precursor rRNA sequences – although the majority of them cleave downstream of U and upstream of ACA nucleobases, some of them prefer an A downstream of the cleavage site and C or G upstream (Figure 2). Furthermore, the recently described MazF-mt9 is specialized for a particular tRNA^*Lys*43–UUU^ species (Barth et al., 2019). VapCs also exhibit specificity for a multitude of different tRNAs or even a tRNA stem-loop structure-mimicking the 23S SRL (Winther et al., 2016) (Figure 4). The AtaT-like toxins that target charged tRNAs are also specific to different tRNAs and even though their toxicity relies on the acetylation of the cargo amino acid charged on its cognate tRNA, these toxins most likely recognize both the amino acids and the tRNA sequence (Jurenas et al., 2017a; Rycroft et al., 2018; Wilcox et al., 2018). Likewise, different HipA toxins have been recently demonstrated to phosphorylate different aminoacyl-tRNA synthetases (Germain et al., 2013; Kaspy et al., 2013; Vang Nielsen et al., 2019). RelE family toxins seem to recognize the mRNA pre-loaded ribosome rather than the particular mRNA sequence. Although some RelE toxins show some preference toward certain nucleobases at certain positions of a codon, the specificity comes not only from the toxin itself, but also from the conformation of ribosomes induced by the binding of these toxins (Neubauer et al., 2009; Feng et al., 2013; Maehigashi et al., 2015; Schureck et al., 2016b; Pavelich et al., 2019). Consequently, the specialization of RelEs involves the evolution of the interactions with ribosomes that in turn direct the substrate recognition.

### Evolutionary Links of Type II TA Toxins

In this review, we have discussed a number of toxin families that inhibit translation at different steps. Not surprisingly, some of these families have potential evolutionary links with proteins involved in the translation maintenance. Toxins targeting RNAs share folds with RNA metabolism associated proteins, in particular those used in RNA maturation and processing (dsRBD, HEPN and PIN-like domains) (Anantharaman and Aravind, 2006; Makarova et al., 2006; Anantharaman et al., 2013). These protein folds are not limited to type II TA toxins, they also take part in other defense and offense systems in prokaryotes as well as in eukaryotes; namely, dsRBD, HEPN, ferredoxin-like, PIN, FIC, and BECR domains can be detected in RNA interference, antitransposon and antiviral systems (Makarova et al., 2006; Kwon et al., 2012; Zhang et al., 2012; Anantharaman et al., 2013). The MazF endoribonucleases comprise a distinct SH3-like barrel-fold rather than a typical nucleic acid-binding domain. The classical SH3 domain is common in eukaryotic cell-to-cell communication and signal transduction proteins, such as signaling kinases, but is less evident in prokaryotes. It has been speculated that bacterial SH3-domain proteins act in eukaryotic cell invasion by corrupting cell signaling (Whisstock and Lesk, 1999). MazF however belongs to the group of proteins that possess domains that structurally resemble SH3, but have diverse functions and enzymatic activities (Whisstock and Lesk, 1999). Therefore, it is not clear whether the SH3 structural motif observed in MazF has a true evolutionary link with other SH3-domain proteins. Similarly, HipA toxins comprise a fold similar to (PI)3/4-kinases found in eukaryotes. These eukaryotic kinases produce 3′ phosphoinositide lipids that bind and activate proteins and therefore participate in signaling cascades (Lempiainen and Halazonetis, 2009). However, a certain class of eukaryotic PI3K family proteins are also Ser/Thr kinases (like HipA toxins) that respond to DNA damage (ATM and DNA-PKcs), nutrient stress (mTOR) or are involved in nonsense-mediated mRNA decay (SMG-1) or transcription regulation (TRRAP) (Lempiainen and Halazonetis, 2009). Whether these eukaryotic proteins have real evolutionary links with HipA toxins remains unclear.

GNAT-fold acetyltransferases are among the most abundant protein folds, however all GNAT-fold TA toxins analyzed to date target the amino group of the amino acid charged on their respective tRNAs (Cheverton et al., 2016; Jurenas et al., 2017a; Wilcox et al., 2018). GNAT-fold acetyltransferases are known to target a wide variety of substrates (Salah Ud-Din et al., 2016). A certain class of GNAT enzymes also acetylate the alpha-amino group of amino acids, however in the context of proteins, i.e., the N-terminal amino acid of peptides after methionine removal. In eukaryotes, this modification is co-translational and affects the majority of proteins, while in bacteria it is limited to specific cases of several ribosomal proteins (Vetting et al., 2008; Favrot et al., 2016). As for GNATs that interact with tRNA, TmcA – an enzyme implicated in translation fidelity – acetylates the wobble cytidine in the anticodon to prevent its misreading (Ikeuchi et al., 2008). Another family of GNATs – the Fem enzymes – use charged tRNAs as substrates for the synthesis of peptidoglycan precursors (Dare and Ibba, 2012). Fic/Doc toxins and secreted toxic effectors generally target eukaryotic and prokaryotic GTPases involved in protein signaling, translation or replication, however, new Fic targets, such as chaperons are emerging (Veyron et al., 2018). Interestingly, three TA toxin folds have also evolved to target topoisomerases (MazF/CcdB, RelE/ParE, Doc/Fic). It is not clear what the link is between translation machinery and topoisomerases. However, topoisomerases are probably the second favorite target of TA toxins. DNA damage caused by these toxins induces a SOS response and DNA repair and could favor rearrangements of genetic material providing higher chances for TAs to relocate. However, it is not clear why the mechanism of choice is blocking topoisomerases. It is worth noting that the translation machinery and topoisomerases are also among the most common targets of antibiotics. Lastly, novel enzymatic activities and targets of TA toxins have been increasingly reported, for example ADP-rybosyltransferase-fold toxins were shown to act by NAD^+^ phosphorolysis and its depletion, or by DNA ribosylation (Jankevicius et al., 2016; Freire et al., 2019). These are the first examples of ADP-ribosyltransferase toxins (ART) involved in TA systems, however, many examples of secreted ART toxins are known and predicted, those also involve translation inhibitors, such as the diphtheria toxin (Zhang et al., 2012).

### What Are the Roles of Type II TA Systems?

The role(s) of TA systems in bacterial physiology and evolution is a long-standing debate (Magnuson, 2007; Tsilibaris et al., 2007; Van Melderen and Saavedra De Bast, 2009; Van Melderen, 2010; Ramisetty et al., 2016; Harms et al., 2017; Culviner and Laub, 2018; Goormaghtigh et al., 2018a, b; Holden and Errington, 2018; Kaldalu et al., 2019; Mets et al., 2019; Pontes and Groisman, 2019; Wade and Laub, 2019; Fraikin et al., 2020). Since their discovery on plasmids in the 1980’s and on chromosomes almost 20 years later, the TA field has been going through waves of hypothesis ranging from replicon maintenance, programmed cell death, stress response, generation of specialized ribosomes, persistence to antibiotics, to phage abortive infection mechanisms. The mainstream hypothesis for the last 10 years was the central role played by type II TA systems in persistence to antibiotics. The hypothesis is that TA systems would be induced in persister subpopulations, thereby stopping their growth and allowing these cells to tolerate the presence of antibiotics. While this hypothesis prevailed for several years, contradicting data accumulated and eventually lead to the retraction of the main papers thereby questioning the involvement of TA systems in drug tolerance (Tsilibaris et al., 2007; Conlon et al., 2016; Ramisetty et al., 2016; Harms et al., 2017; Shan et al., 2017; Goormaghtigh et al., 2018a, b; Kaldalu and Tenson, 2019; Pontes and Groisman, 2019). Although TA systems are occasionally found upregulated under stress conditions in transcriptomic data (Keren et al., 2004), this could be a natural consequence of de-repression of TA loci due to instability and degradation of the antitoxins. Since TA systems present selfish behavior, it is tempting to compare them to viral elements, and to look at TAs from the perspective of genes, and not of the organism. In this review we have provided a detailed view of the specialization of toxins sharing the same fold. Such a specialization seems to be the general trend for type II TA toxins and is seen in virtually all families that have at least several studied examples. If such systems would be part of a stress-response system, however, one would expect the selection and conservation of the ‘best’ mode of action. On the contrary, the reservoir and activities of TA systems from different strains is highly variable and reminds the variety seen in offense and defense systems that are used for competition between species where innovations are beneficial. Among the driving forces for the evolution of different substrate specificities could be the tight neutralization of each toxin by its cognate antitoxin. Co-evolution of different antitoxins and different toxins relies on their vast contacts, necessary for neutralization and transcriptional autoregulation. This dependency should in addition allow for a faster evolution and selection of changes. Further, the competition between an incoming near-identical TA system likely provides selective pressure. The incoming TAs, if identical, would be neutralized by an existing copy of the TA system and such an ‘anti-addiction’ module therefore would prevent stable establishment of identical TAs (Van Melderen and Saavedra De Bast, 2009). Indeed, it has been shown that in some cases the different TAs in the same organism are only insulated by 1 amino acid difference (Walling and Butler, 2018), indicating that a strong selection might apply on TAs to avoid cross-talks. Lastly, the high abundance of TA systems on mobile genetic elements supports the idea that TA systems are primarily elements involved in intergenomic conflicts – inheritance of existing, defense against incoming and offense or spread of new genetic material.

## Author Contributions

All authors listed have made a substantial, direct and intellectual contribution to the work, and approved it for publication.

## Conflict of Interest

The authors declare that the research was conducted in the absence of any commercial or financial relationships that could be construed as a potential conflict of interest.
